# Non-structural carbohydrates and morphological traits of leaves, stems and roots from tree species in different climates

**DOI:** 10.1186/s13104-022-06136-7

**Published:** 2022-07-15

**Authors:** Guangqi Zhang, Pascale Maillard, Zhun Mao, Loic Brancheriau, Julien Engel, Bastien Gérard, Claire Fortunel, Jean-Luc Maeght, Jordi Martínez-Vilalta, Merlin Ramel, Sophie Nourissier-Mountou, Stéphane Fourtier, Alexia Stokes

**Affiliations:** 1 AMAP, Université de Montpellier, CIRAD, CNRS, INRAE, IRD, 34000 Montpellier, France; 2 SILVA, INRAE, Université de Lorraine, Agroparistech, Centre de Recherche Grand-Est Nancy, 54280 Champenoux, France; 3 CIRAD, Université de Montpellier, UR BioWooEB, 34000 Montpellier, France; 4 CREAF, Bellaterra, 08193 Cerdanyola del Vallès, Catalonia Spain; 5 Universitat Autònoma Barcelona, Bellaterra, 08193 Cerdanyola del Vallès, Catalonia Spain

**Keywords:** Angiosperms, Non-structural carbohydrates, Fibers, Functional traits, Mediterranean, Parenchyma, Root, Temperate, Tropical, Vessels

## Abstract

**Objectives:**

Carbon fixed during photosynthesis is exported from leaves towards sink organs as non-structural carbohydrates (NSC), that are a key energy source for metabolic processes in trees. In xylem, NSC are mostly stored as soluble sugars and starch in radial and axial parenchyma. The multi-functional nature of xylem means that cells possess several functions, including water transport, storage and mechanical support. Little is known about how NSC impacts xylem multi-functionality, nor how NSC vary among species and climates. We collected leaves, stem and root xylem from tree species growing in three climates and estimated NSC in each organ. We also measured xylem traits linked to hydraulic and mechanical functioning.

**Data description:**

The paper describes functional traits in leaves, stems and roots, including NSC, carbon, nitrogen, specific leaf area, stem and root wood density and xylem traits. Data are provided for up to 90 angiosperm species from temperate, Mediterranean and tropical climates. These data are useful for understanding the trade-offs in resource allocation from a whole-plant perspective, and to better quantify xylem structure and function related to water transportation, mechanical support and storage. Data will also give researchers keys to understanding the ability of trees to adjust to a changing climate.

## Objective

Non-structural carbohydrates (NSC) are essential substrates for metabolic processes in trees, including respiration, osmoregulation, growth, reproduction and defense [1–4], as well as having major consequences for downstream processes such as microbial activity in the rhizosphere [5]. NSC is a product of photosynthesis and comprises mainly soluble sugars involved in transport or immediate functions, and starch stored in different plant organs for future use and maintaining functionality when carbon demand is higher than supply (e.g., under severe drought stress) [6–9]. Therefore, understanding how patterns of NSC vary in trees will enable us to better evaluate the role of NSC in tree physiological processes and ecological strategies, especially across a broad range of species and climates [10].

The secondary xylem of angiosperms is generally composed of three specialized cell types including parenchyma, vessels and fibers (tracheids and fiber-tracheids may also be present), that perform storage, hydraulic and mechanical functions [11–13]. NSC are mainly stored in the live radial and axial parenchyma cells, therefore the size of the parenchyma fraction drives the capacity for NSC storage in trees [14–16]. In an attempt to disentangle how NSC and xylem traits are linked to tree physiological processes and ecological strategies, we collated data on stem and root NSC and xylem cell patterns, as well as leaf traits, from 90 tree species in temperate, Mediterranean and tropical climates. Our data will allow researchers to explore the direct relationships between leaf, stem and root NSC contents with patterns in xylem cell composition. To our knowledge, this dataset represents the largest freely available collection of data for NSC xylem and leaf traits measured simultaneously in adult trees from diverse climates. Detailed information on materials and methods can be found in the accompanying Excel file (Table 1).

**Table 1:** Overview of data files/data sets

Label	Name of data file/data set	File types (file extension)	Data repository and identifier (DOI or accession number)
Data file 1	Xylem_Stem_Root_Leaf_Trait data	MS Excel file (.xlsx)	Portail Data INRAE 10.15454/MU0HXX [23]

## Data description

### Study sites and species

This study was conducted in a temperate forest (Luz-Saint-Sauveur, France), a Mediterranean forest (Montpellier, France) and a tropical forest (Paracou, French Guiana). A total 90 angiosperm species were collected which are commonly found in local forests, with 20 species in both temperate and Mediterranean climates and 50 species in the tropical climate (Table 1, file 1). For each species, we chose three, healthy, adult trees that usually had a stem diameter of between 0.05 and 0.4 m at a height of 1.3 m. We collected leaf and stem samples for all trees, as well as coarse root samples for 60 species (n = 20 in each climate). Samples were collected at the end of August and early September 2019 for Mediterranean and tropical species and September 2020 for temperate species, when NSC storage should be close to its seasonal maximum [17–19]. We sampled all trees between 7am and midday, to reduce variability linked to photosynthate production. At a height of 1.3 m, three 0.05 m long cores were extracted from tree stems with a 4.3 mm diameter increment borer. To collect samples of roots, we excavated a single lateral root (0.02–0.05 m in diameter) and at a distance of 0.3–0.5 m from the base of the tree and extracted three increment cores or removed three 0.02 m long segments of root. We also collected one stem core from each tree in temperate and Mediterranean climates in March 2021 (before bud burst). In total, 540 leaf samples, 930 stem cores and 540 root segments were collected.

### Measurement of leaf and xylem functional traits

A total of 270 leaf samples, 390 stem samples and 180 root samples were used to determine NSC, carbon (C) and nitrogen (N) measurements. For NSC content, a colorimetric method [20] was performed on all leaf samples, and a subsample of stems (n = 113) and roots (n = 140). C and N content were measured in a sub-sample of leaves (n = 110), stems (n = 113) and all root samples, using an elemental analyzer (CHN model EA 1108, Milan, Italy). Then, near infra-red spectroscopy (NIRS) [21] was performed on all samples and calibration models developed using data obtained from the analytical methods to predict soluble sugars, starch, C and N in the three organs (Table 1, file 1).

Cross-sections of 15–20 µm thick for stem (n = 270) and root (n = 180) samples were cut with a sliding microtome and stained with a mixture of safranin and alcian blue. Microphotographs of transversal sections were taken where radial and axial parenchyma fractions, vessel fractions, mean vessel area, mean vessel diameter, vessel density, mean vessel hydraulic diameter and theoretical specific xylem hydraulic conductivity were determined [22] (Table 1, file 1).

Specific leaf area (n = 270) was determined using leaf area and dry mass. Stem wood density (n = 270) and root wood density (n = 180) were defined as oven-dry mass divided by fresh volume which measured by water displacement method (Table 1, file 1).

## Limitations

Root NSC data were only collected for 60 species (n = 20 in each climate type). It was not possible to collect NSC in samples from tropical trees over two different seasons within a 12-month time period because of travel restrictions linked to the COVID pandemic. However, there is significantly less seasonal variability in NSC in tropical trees [10], compared to Mediterranean and temperate species, therefore we do not consider this limitation as detrimental to the quality of data.

## Data Availability

The data and methods described in this Data note can be freely and openly accessed on Portail Data INRAE under https://doi.org/10.15454/MU0HXX. Please see Table 1 and reference [23] for details and links to the data.

## Abbreviations

NSC: Non-structural carbohydrates
C: Carbon
N: Nitrogen
NIRS: Near infra-red spectroscopy
